# Biodiversity, Phylogeny, and Antifungal Functions of Endophytic Fungi Associated with *Zanthoxylum bungeanum*

**DOI:** 10.3390/ijms17091541

**Published:** 2016-09-13

**Authors:** Peiqin Li, Zhou Wu, Tao Liu, Yanan Wang

**Affiliations:** 1 Department of Forest Protection, College of Forestry, Northwest A&F University, Yangling 712100, China; 15229371653@163.com (Z.W.); lt937215187@163.com (T.L.); 2 Department of Landscape Architecture, College of Landscape Architecture and Arts, Northwest A&F University, Yangling 712100, China; wang55531@126.com

**Keywords:** *Zanthoxylum bungeanum*, endophytic fungi, fungal diversity, phylogeny, antifungal activity

## Abstract

This study investigated the biodiversity, phylogeny, and antifungal activity of endophytic fungi isolated from *Zanthoxylum bungeanum*. A total of 940 isolates obtained were grouped into 93 morphotypes, 43 species, and 23 genera, which were authenticated by molecular identification based on rDNA internal transcribed spacer (ITS) sequence analysis. A high diversity of endophytic fungi from *Z. bungeanum* are observed with high species richness *S* (43), Margalef index *D*′ (6.1351), Shannon–Wiener index *H*′ (3.2743), Simpson diversity index *D*_s_ (0.9476), *PIE* index (0.9486), and evenness Pielou index *J* (0.8705) but a low dominant index *λ* (0.0524). Significant tissue specificity of the endophytic fungi was observed in *Z. bungeanum*, and the highest species richness and diversity indexes were obtained in the stem. Phylogenetic analyses of the 93 endophytic isolates were carried out by the neighbor-joining (NJ) method to demonstrate their evolutionary processes. Antifungal activities of endophytic fungi were assayed and eight endophytic isolates showed strong and long-lasting inhibition against host pathogenic fungi *Fusarium sambucinum* and *Pseudocercospora zanthoxyli*. Here, for the first time, we systematically demonstrate the biodiversity, phylogeny, and antifungal activity of endophytic fungi associated with *Z. bungeanum* and reveal the value of sampling different tissues of a given plant to obtain the greatest endophyte species diversity, which might offer a framework for further investigation and utilization of endophytic fungi as aunique source of interesting and useful bioactive compounds.

## 1. Introduction

Endophytic fungi have been reported as novel sources of bioactive compounds to be applied in the agricultural field. It has been frequently reported that endophytic fungi can protect host plants against pests by producing protective compounds, conferring the resistances of host plants to biotic or abiotic stresses by enhancing defensive system and improving the growth and product yield of plants directly or indirectly [1,2,3]. Given the sideeffects of indiscriminate use of conventional chemical fungicides, i.e., contaminating environment, damaging human health, inducing pathogen resistance to fungicides, and causing resurgence of plant disease, the exploitation of natural bio-control agents has become an overwhelming trend in integrated pest management [4,5,6]. Plant endophytic fungi are just such natural resources of bio-control agents. Substantial renewed attention has been paid to the inhibitory activity of endophytic fungi against pathogenic fungi and their potential in the biological control of plant diseases [7,8].

*Zanthoxylum bungeanum* is an aromatic plant of the family Rutaceae and is native to southwestern China. It has a long history as a pungent foodstuff and seasoning in Korea, China, and other East Asian countries [9]. Phytochemical studies of *Z. bungeanum* have been carried out in recent years including compound isolation, structure elucidation, extraction of essential oils and its pharmacological activities, and extraction optimization of polysaccharide and antioxidant activities, which demonstrated the importance of this plant due to its huge economic value [10,11,12,13]. However, different pathogenic plant fungi frequently infect *Z. bungeanum* during its growth process, which causes serious effects on yield and quality [14]. The most reported pathogenic fungi isolated from *Z. bungeanum* were *Pseudocercospora zanthoxyli* and *Fusarium sambucinum* in the Shaanxi and Gansu districts, which resulted in prickly ash leaf mold and stem dry rot, respectively [15,16]. The use of chemical pesticides is the current main method of controlling the aforementioned pathogenic fungi [17]. Considering the sideeffects of chemical fungicides, it is necessary to explore new nontoxic and efficient alternatives to synthetic pesticides to control plant pathogenic fungi.

Until recently, there have been few reports about the endophytic fungi of *Z. bungeanum*. One study about endophytic fungi from pericarpium zanthoxyli was reported by Liu et al. [18], in which 12 endophytic fungal isolates were obtained from pericarpium zanthoxyli and found one isolate that could produce a volatile, fragrant metabolite. However, the endophytic fungi from pericarpium zanthoxyli in this report were not identified, and the variety and development stage of zanthoxyli were also not introduced clearly, both of which are considered important factors affecting the number and species of endophytic fungi obtained from plant tissues [18,19]. Hence, it is critical to conduct systematic studies on the biodiversity analysis and antifungal activities of endophytic fungi from *Z. bungeanum*. The objectives of this research were to explore the α diversity and phylogenetic relationships of endophytic fungi associated with *Z. bungeanum* different tissues and to screen for isolates with obvious antifungal activity against the pathogenic fungi *P. zanthoxyli* and *F. sambucinum*. Our present research is aimed at further investigating the evolution of endophytic fungi communities in plant micro-ecological systems and providing valuable information for the exploitation of effective natural bio-control agents for *Z. bungeanum* diseases.

## 2. Results

### 2.1. Identification of Endophytic Fungi from Z. bungeanum

As shown in Table 1, a total of 940 isolates were obtained from *Z. bungeanum*. Specifically, 286 isolates originated from the stem, 264 from the roots, 202 from leaves, 106 from the fruit, and 82 from thorns. The isolates from different tissues were separated, even though they possessed the same morphological characteristics. According to their morphological characteristics, the isolates from the roots, stem, leaves, fruit, and thorns were preliminarily categorized according to morphotypes, which were recorded as Zbf-R, Zbf-S, Zbf-L, Zbf-F, and Zbf-T, respectively. The numbers of morphotypes were determined as: 15 in the roots, 50 in the stem, 11 in leaves, nine in fruit, and eight in the thorns. In total, 93 isolates representing 93 morphotypes were molecularly identified. As shown in Table 1, DNA fragments generated by polymerase chain reaction (PCR) amplification of the internal transcribed spacer (ITS) rDNA region ranged from 500 to 650 bp in size and were subsequently sequenced. By analyzing their ITS rDNA regions using the basic local alignment search tool (BLAST) in the National Center for Biotechnology Information(NCBI) GenBank, a list of identification results of endophytic fungi isolates with their GenBank accession number in NCBI, the closest species, accuracy (query coverage), and genetic similarity to the sequences deposited (identity) were summarized (Table 1). For the majority of endophytic isolates, the genetic identities are 99% and 100%. The only exceptions are Zbf-S38 *Phomopsis vaccinii* (97% identity) and Zbf-T3 *Diaporthe cotoneastri* (98% identity). The ITS sequences of these 93 endophytic fungal isolates and their taxonomic identifications have been deposited in GenBank.

### 2.2. Clustering and Phylogenetic Analyses of Endophytic Fungi

As presented in Table 2, the majority of endophytic fungi isolated from *Z. bungeanum* are included in the Ascomycota (910 isolates) and within three classes: Sordariomycetes, Dothideomycetes, and Eurotiomycetes. There are only 30 isolates clustered in the Basidiomycota within the Agaricomycetes. The dominant group of endophytic fungi is the Dothideomycetes (63.30%, 20 species), which contains Pleosporales (55.85%, eight species) and Botryosphaeriales (7.45%, two species). It is followed by Sordariomycetes (32.77%, 19 species), which includes Hypocreales (19.68%, 10 species), Diaporthales (11.49%, eight species) and Xylariales (1.60%, one species). There are only two species of endophytic fungi belonging to the Eurotiomycetes Eurotiales (0.74%), *Aspergillus flavus* and *A. fumigates*. The endophytic fungi categorized in the Agaricomycetes (3.19%, two species) include Polyporales *Irpex lacteus* (0.32%) and Auriculariales *Auricularia polytricha* (2.87%). In total, 43 species of endophytic fungi, distributed through 23 genera, were obtained from sampling *Z. bungeanum*.

**Figure 1:**
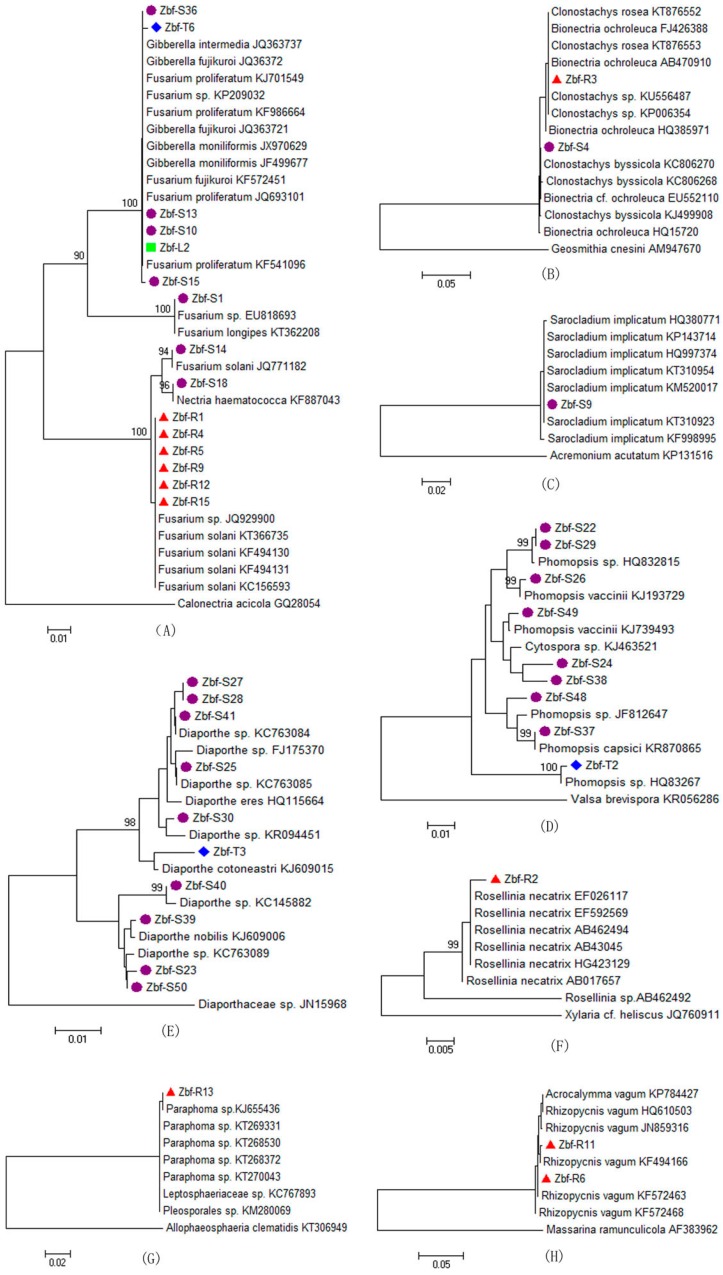

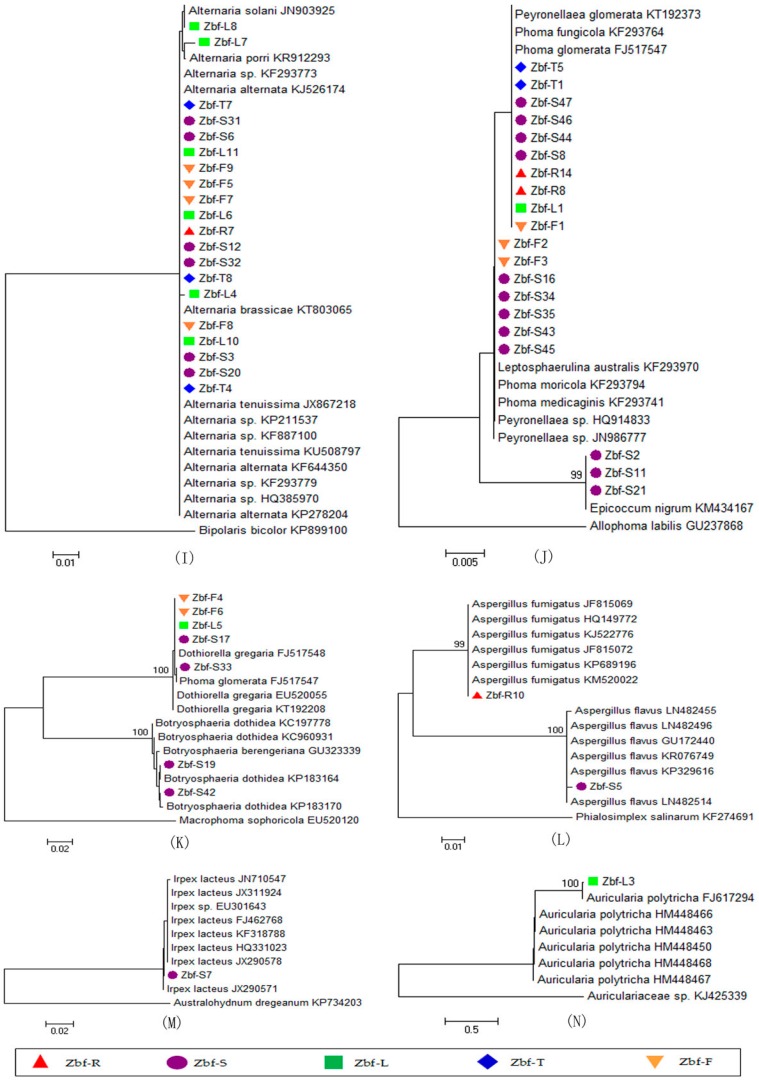
Neighbor-joining phylogenic analyses by internal transcribed spacer (ITS) sequence alignment for the endophytic fungi from *Z. bungeanum* belonging to Nectriaceae (**A**); Bionectriaceae (**B**); Valsaceae (**C**); Diaporthaceae (**D**); Xylariaceae (**E**); Incertae sedis Hypocreales (**F**); Pleosporaceae (**G**); Didymellaceae (**H**); Phaeosphaeriaceae (**I**); Morosphaeriaceae (**J**); Botryosphaeriaceae (**K**); Aspergillaceae (**L**); Incertae sedis Polyporales (**M**); and Auriculariaceae (**N**).

To confirm the identification and clustering results, phylogenetic analyses of 93 endophytic fungi were carried out by comparing their ITS sequences with their closest species from NCBI using the neighbor-joining (NJ) method. All of the endophytic fungi obtained from *Z. bungeanum* were clustered in 14 families, which were marked by capital letters A to N (Table 2). The isolates belonging to the same family were clustered in the same phylogenetic tree. Additionally, sequences with the closest relationship to each analyzed isolate were also acquired from GenBank to demonstrate the phylogeny of these endophytic fungi. Each NJ tree includes one outlying group belonging to the same family but a different genus to confirm the endophytic fungi phylogenetic placements. Figure 1 presents 14 NJ phylogenetic trees, in which the bootstrap values less than 90% were not shown in the NJ treeclades. Figure 1A–N represents the NJ trees of the families Nectriaceae, Bionectriaceae, Valsaceae, Diaporthaceae, Xylariaceae, Incertae sedis Hypocreales, Pleosporaceae, Didymellaceae, Phaeosphaeriaceae, Morosphaeriaceae, Botryosphaeriaceae, Aspergillaceae, Incertae sedis Polyporales, and Auriculariaceae, respectively. The endophytic fungal phylogenetic trees verified their taxonomic positions, which were overall correct at the genus level.

As shown in Figure 1, the endophytic fungal phylogenetic clustering is consistent with their identification at the species level. Although the isolates Zbf-S36, Zbf-T6, Zbf-S13, Zbf-S10, Zbf-L2, and Zbf-S15 were not accurately correlated to their corresponding species (Figure 1A), this is not surprising because *Gibberella* is the teleomorph of *Fusarium*. Besides, the anamorph phases of *Bionectria*, *Diaporthe*, and *Botryosphaeria* are *Clonostachys*, *Phomopsis*, and *Dothiorella*, respectively [20,21,22]. Based on a genetic standpoint, fungi with different generic names for the teleomorph and anamorph are actually the same fungus. However, we do not combine the teleomorph and anamorph of the fungus for their different morphological characterization.

### 2.3. Relative Abundance Analyses of Endophytic Fungi from Z. bungeanum

**Figure 2:**
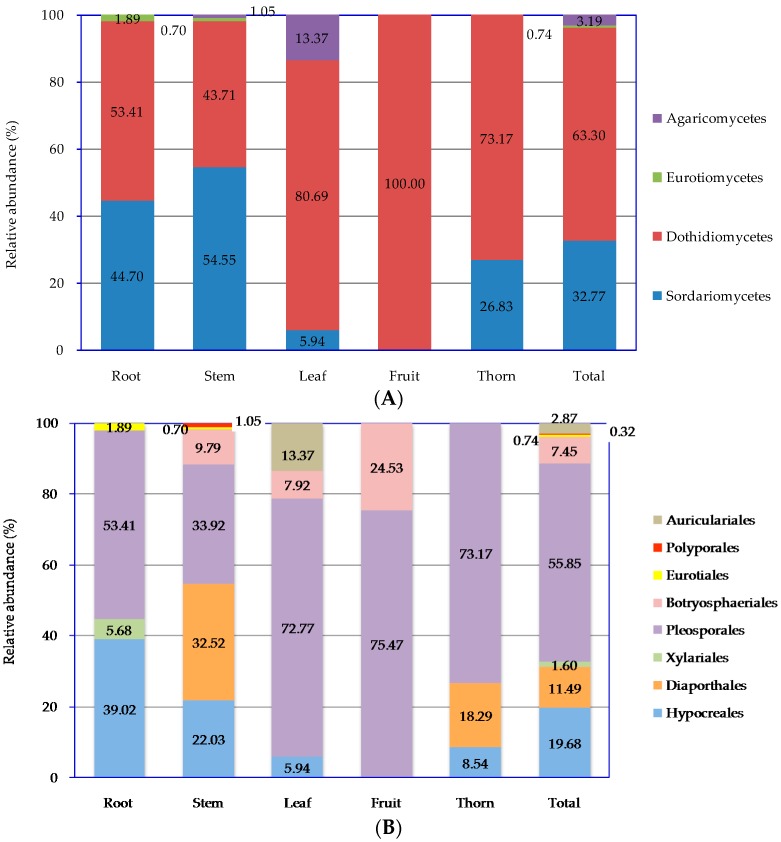
Relative abundance (%) of endophytic fungal at the level of class (**A**); and order (**B**) in different tissues and total plant of *Z. bungeanum*.

Numerous culturable endophytic fungal isolates were obtained from five different *Z. bungeanum* tissues, all of which were classified in two phyla, four classes, eight orders, 14 families, 23 genera, and 43 species. At the phylum level, there were no obvious differences among the tissues because all of the endophytic fungal isolates belonged to Ascomycota except for Zbf-S7 and Zbf-L3 (Table 2). Ascomycota was the main phylum found in *Z. Bungeanum* isolates. There was a low proportion of Basidiomycota endophytic fungi in the *Z. bungeanum* stem and leaves. The relative abundance (RA, %) of endophytic fungi at the levels of class and order are presented in Figure 2.

As shown in Figure 2A, Dothideomycetes was the main community member, although it showed a distinctly different relative abundance in each tissue. Dothideomycetes accounted for 53.41% in the roots, 43.71% in the stems, 80.69% in leaves, 100% in the fruits, and 73.17% in thorns. Sordariomycetes was the second main community member. Figure 2B presented the relative abundance of endophytic fungi at the order level. There were eight orders endophytic fungi from *Z. bungeanum* in total. Among the plant tissues examined, stems hosted the most fungal orders (six orders). Pleosporales was the most dominant community in every tissue with the highest RA compared with other orders. The relative abundances of Pleosporales were 53.41% in the roots, 33.92% in the stems, 72.77% in the leaves, 75.47% in the fruits, and 73.17% in the thorns.

**Figure 3:**
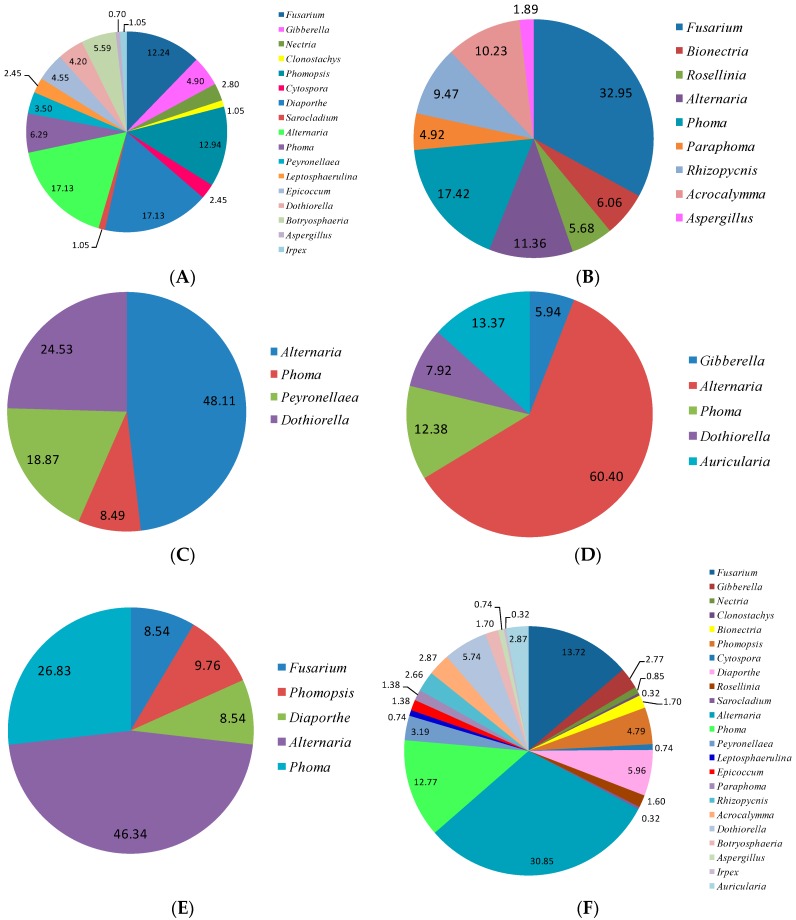
Relative abundance (RA, %) of endophytic fungal at the level of genera in stem (**A**); root (**B**); fruit (**C**); leaf (**D**); thorn (**E**); and total plant (**F**) of *Z. bungeanum*.

The relative abundance of endophytic fungi genus in different *Z. bungeanum* tissues was analyzed in Figure 3. In total, 23 genera of endophytic fungi were observed for the entire *Z. bungeanum* plant. The stems possessed the highest genus richness with 17 genera, followed by the roots with nine genera, leaves with five genera, thorns with five genera, and the fruits with four genera. *Alternaria* and *Phoma* genera were observed in all tissues of *Z. bungeanum*, and the predominant genera were *Alternaria* (30.85%), *Fusarium* (13.72%), and *Phoma* (12.77%). The genus *Auricularia* was specific to the leaves. There were five genera specific to the roots, which were *Bionectria*, *Rosellinia*, *Paraphoma*, *Rhizopycnis*, and *Acrocalymma*. For the stems, there were six specific genera, *Nectria*, *Clonostachys*, *Sarocladium*, *Leptosphaerulina*, *Epicoccum*, and *Irpex*.

### 2.4. Diversity Analyses of Endophytic Fungi from Z. bungeanum

Table 3 summarizes the species of endophytic fungi in every tissue, the number of isolates (*N*) and isolation frequency (*IF*) for each species in detail. There were 11 species of endophytic fungi in the root, 23 species in the stem, nine species in the leaves, six species in the fruit, and seven species in the thorns. Endophytic fungi species showed obvious dispersive and specific distribution in different *Z. bungeanum* tissues. Some species were observed in two or more different tissues, and some species were only isolated from one tissue. There were only two species, *Alternaria* sp. and *Phoma medicaginis*, observed in all five tissues. It was concluded from Table 3 that the endophytic fungi species colonizing in different tissues were various, and the isolation frequency of each species was varied depending on the tissue colonized.

The diversity indices of endophytic fungi species associated with *Z. bungeanum* are summarized in Table 4. The species richness (*S*) and Margalef index (*D*′) can reflect the richness of endophytic fungi species. The larger the values of S and D′ are, the richer the species of endophytic fungi are [23]. The species diversity can be analyzed by the Shannon–Wiener index (*H*′), Simpson diversity index (*D*_s_), and probability of interspecific encounter index (*PIE*). These indices take into account the heterogeneity/homogeneity of the species frequencies. Generally, the higher the Shannon’s diversity index (commonly ranging between 1.5 and 4.5) and the closer the Simpson’s diversity index is to 1, the more intensified heritable variation and stronger adaptive capacity for micro-environmental change the communities presented as they tended to expand the distribution range and enter new environments. As shown in Table 4, endophytic fungi colonizing the stem showed the highest species richness and diversity, with maximum values of *S* (32), *D*′ (5.4809), *H*′ (3.2010), *D*_s_ (0.9502), and *PIE* (0.9536). The varied trends of *H*′, *D*_s_ and *PIE* should be kept consistent. However, there were slight differences for the endophytic fungi from the roots, leaves, fruit, and thorns, which might be attributed to the significant interaction of the number, isolation frequency, and species richness of the isolates.

The Pielou index (*J*) can reflect the evenness of species, which was evaluated on the basis of the Shannon–Wiener index (*H*′) and the size of samples. In this study, the endophytic fungi from thorns showed the highest Pielou index, even though it had a relatively lower species richness and Shannon–Wiener index compared with those from the roots, stem, or leaves. The dominant index (*λ*) was used to evaluate the ecological dominance of a community, which was inversely related to Simpson’s diversity index (*D*_s_). If a higher *λ* is observed in the community, it indicates that the community might have low species diversity and evenness. The endophytic fungal community in the *Z.* *bungeanum* stem showed the lowest degree of ecological dominance, with a *λ* value of 0.0498. Overall, endophytic fungal communities from different parts of *Z. bungeanum* showed different species structure, richness, diversity, and dominance. Taking all of the endophytic fungi from the five different tissues together, it could represent the endophytic fungal community of *Z. bungeanum* as a whole. As shown in Table 4, the total endophytic fungi associated with *Z. bungeanum* showed high species richness and diversity but low degrees of ecological dominance with high values of *S* (43), *D*′ (6.1351), *H*′ (3.2743), *D_s_* (0.9476), and *PIE* (0.9486), and low values of *λ* (0.0524).

**Figure 4:**
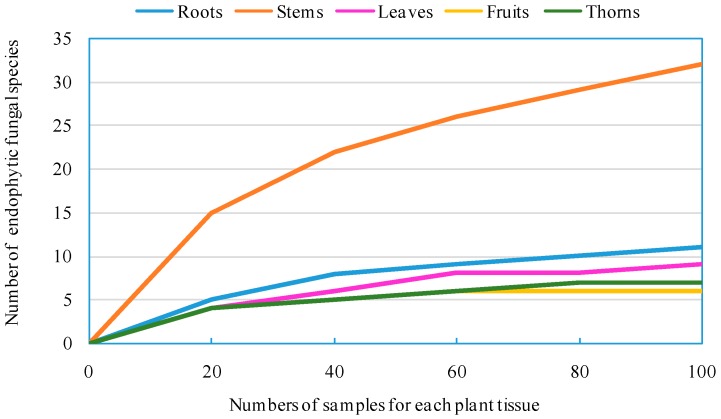
Rarefaction curves of endophytic fungal species for different tissues of *Z. bungeanum*.

Figure 4 showed the rarefaction curves for respective tissues of *Z. bungeanum* using endophytic fungal species as the operational taxonomic unit (OTU). Rarefaction curves can reflect in a straightforward way how the number of fungal species increases with the increasing number of plant tissues. As presented in Figure 4, the increasing number of samples for the stems of *Z. bungeanum* could affect the number of endophytic fungal species.

### 2.5. Antifungal Activities of Endophytic Fungi from Z. bungeanum

The ethyl acetate (EtOAc) extracts of 93 endophytic fungal isolates from *Z. bungeanum* were prepared and their antifungal activities against pathogenic fungi *F. sambucinum* and *P. zanthoxyli* were evaluated in vitro using the radial growth method on the 7th day of culture. The concentrations of EtOAc extracts of endophytic fungi in PDA are summarized in Table 5.

**Figure 5:**
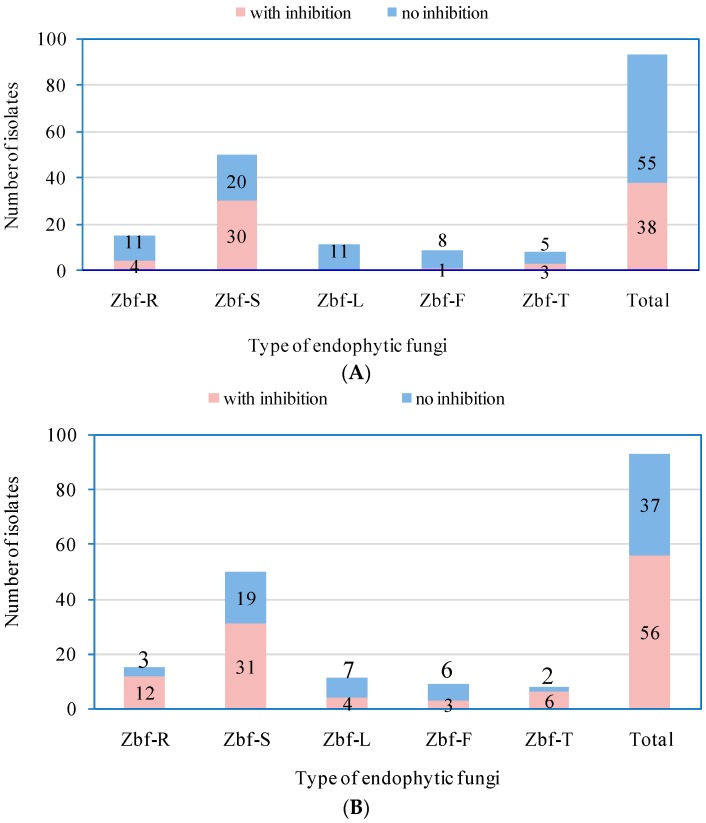
The summarized numbers of endophytic fungal isolates with inhibition and without inhibition against the pathogenic fungi *F. sambucinum* (**A**); and *P. zanthoxyli* (**B**) of *Z. bungeanum*. The values presented in the graph are the corresponding numbers of endophytic isolates with inhibition or without inhibition.

We summarized the numbers of endophytic fungal isolates with inhibition and no inhibition by observing the colony diameter visually and calculating the inhibitory rate (*IR*). As presented in Figure 5A, 38 out of 93 fungal endophytes exhibited inhibition against *F. sambucinum*, which included four isolates from the roots, 30 isolates from the stems, one isolate from the fruits, and three isolates from the thorns. For *P. zanthoxyli*, 56 endophytic fungal isolates showed inhibitory effects, which included 12 isolates from the root, 31 isolates from the stem, four isolates from the leaves, three isolates from the fruit, and six isolates from the thorns (Figure 5B). It was concluded from Figure 5 that more than half of the endophytic fungal isolates from the stems possessed inhibition against both pathogenic fungi, while the endophytic fungi from the leaves showed a low percentage of inhibitory isolates.

**Figure 6:**
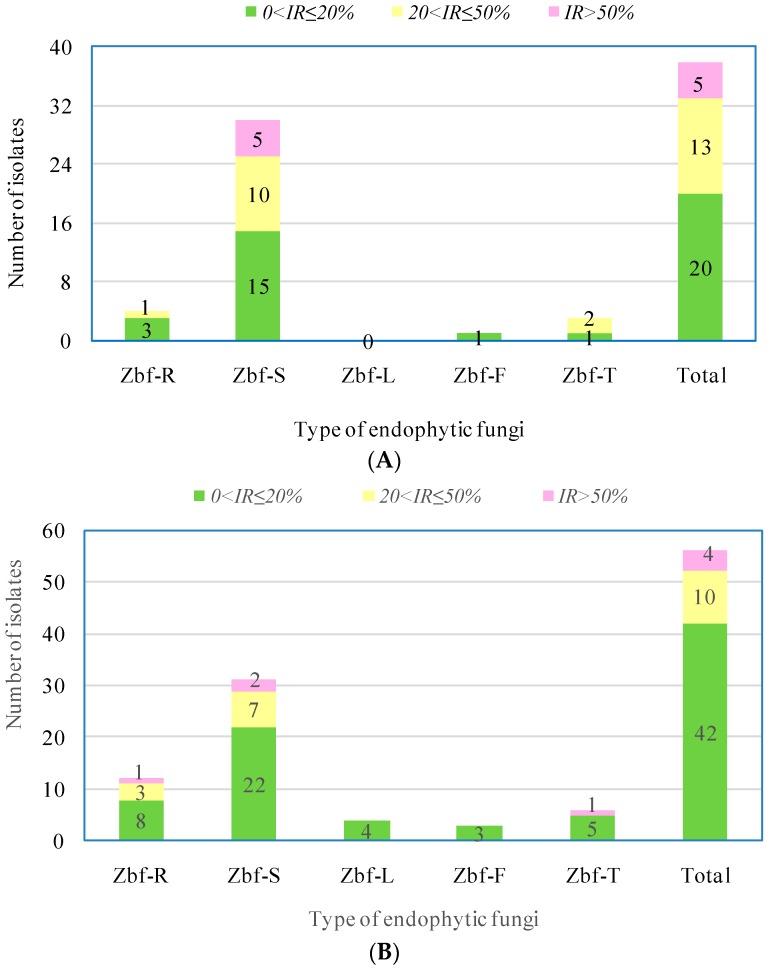
Grading of inhibitory ability of endophytic fungal isolates with inhibition on pathogens *F. sambucinum* (**A**); and *P. zanthoxyli* (**B**). The values presented in the graph are the corresponding numbers of endophytic isolates with inhibition respectively locating their corresponding grades.

We graded the inhibitory rate of all endophytic fungal isolates using three ranges of IR, i.e., 0 < *IR* ≤ 20%, 20% < *IR* ≤ 50%, and *IR* > 50%. As presented in Figure 6, most isolates showed weak inhibitory effects on *F. sambucinum* and *P. zanthoxyli* because their *IR* values were lower than 20%. There were 13 endophytic fungal isolates that showed medium inhibitory effects on *F. sambucinum* with IR values in the range of 20% < *IR* ≤ 50%, which included one isolate from the roots, 10isolates from the stems, and two isolates from thorns. There were five endophytic fungal isolates showing strong inhibition against *F. sambucinum* with *IR* > 50%, all of which were isolated from the stems (Figure 6A). As shown in Figure 6B, there were only 10endophytic fungal isolates that showed medium inhibition against *P. zanthoxyli* with *IR* values in the range of 20% < *IR* ≤ 50%, which included three isolates from the roots and seven isolates from the stem. Four endophytic fungal isolates exhibited strong inhibition against *P. zanthoxyli* with *IR* > 50%, which included one isolate from the roots, two isolates from the stem, and one isolate from the thorns. Altogether, endophytic fungi from the *Z.* *bungeanum* stem showed relatively stronger inhibition against pathogenic fungi of the host and with higher numbers of isolates with strong inhibition.

**Figure 7:**
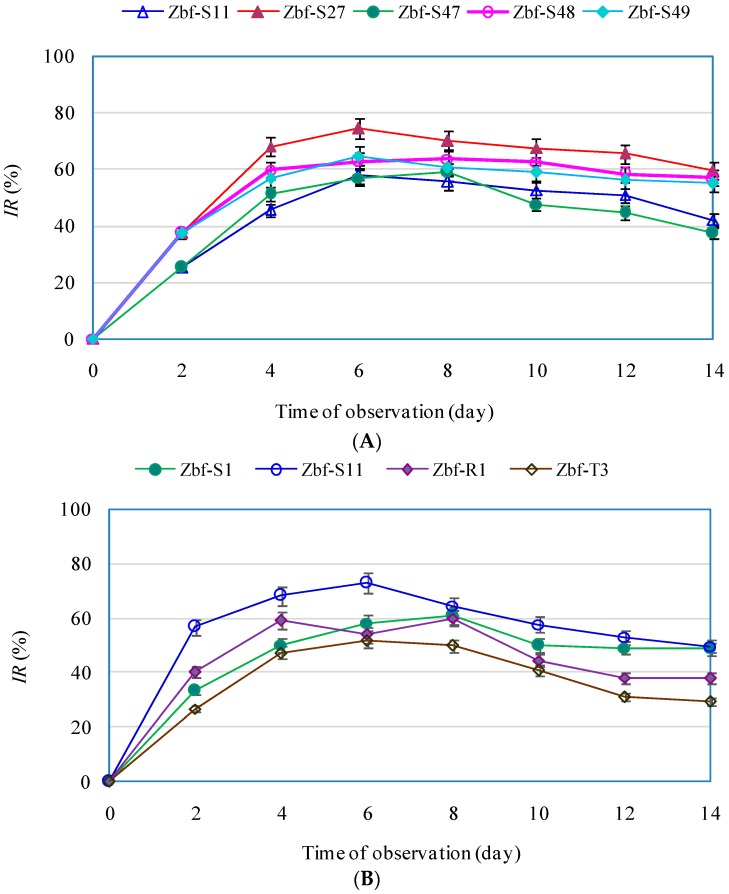
Time dynamics of inhibition rate for the endophytic fungal isolates with *IR*>50% against pathogenic fungi *F. sambucinum* (**A**); and *P. zanthoxyli* (**B**).

The endophytic fungi isolates with *IR* > 50% for *F. sambucinum* were Zbf-S11, Zbf-S27, Zbf-S47, Zbf-S48, and Zbf-S49, and for *P. zanthoxyli* they were Zbf-S1, Zbf-S11, Zbf-R1, and Zbf-T3. Time dynamics of their *IR* values were further investigated on the culture days 2, 4, 6, 8, 10, 12, and 14. As presented in Figure 7A, the inhibition rates of all five endophytic isolates against *F.* *sambucinum* increased linearly from the beginning of culture to the fourth day, reached their maximums on the sixth day, declined slightly and then remained at high, stable levels until the 14th day with *IR* ≥ 40%. It was concluded that the EtOAc extracts of all of the five endophytic fungi from the stems of *Z. bungeanum* possessed long lasting efficacy. The isolate Zbf-S27 showed the strongest inhibition effect during the entire culture period with its maximum *IR* 74.32% on the sixth day and had a high *IR* value of 59.60% even on the 14th day. For *P. zanthoxyli*, shown in Figure 7B, Zbf-S11 showed the strongest inhibition effect, and its inhibition rate was always higher than those of other isolates during all of the culture days. The maximum *IR* 72.81% of Zbf-S11 on *P. zanthoxyli* was observed on the sixth day. These eight endophytic fungi isolated from *Z. bungeanum* have the potential for inhibiting the growth of pathogenic fungi and their antimicrobial compounds are worth further investigation.

## 3. Discussion

Plant endophytic fungi are highly taxonomically diverse and are also demonstrated to adjust the morphological and physiological functions of the host plant through multiple mechanisms, including stimulating its resistance to biotic and abiotic stresses [24]. It is important to explore endophytic fungi from different plants to obtain many natural resources as well as to understand the biodiversity of endophytic fungal community in a symbiotic relationship. Considering the fragrant specificity of *Z. bungeanum*, there is a possibility to isolate novel endophytic fungi that possess special functions. Isolating and identifying new endophytic fungi from *Z. bungeanum* might lead to the discovery of new and unusual compounds with biotechnological and pharmaceutical applications. In our present study, 940 endophytic fungal isolates were obtained from the roots, stems, leaves, fruits, and thorns of *Z. bungeanum* by a culture-dependent method, which were subsequently categorized into 93 morphotypes, 43 species, 23 genera, eight orders, four classes, and two phyla. A high number of endophytic fungal species were encountered during this relatively small survey, despite the fact that the methodology employed in our research is culture-specific and slow growing, and some non-culturable species are likely to be missed [25]. Diversity analysis shows that endophytic fungi residing in *Z. bungeanum* are highly diverse. It has been widely reported that culture-dependent methods for isolating microbes from surface-sterilized plant tissues result in a large quantity of endophytic fungi, and researchers have analyzed their biodiversity [24,26]. Specifically, the endophytic fungi from the roots, stems, leaves, fruits, and thorns of *Z. bungeanum* were separately preserved and identified, which exhibited obvious tissue specificity. The endophytic fungi tissue specificity in different parts of *Z. bungeanum* may be caused by differences in the plant tissue microenvironment. Host plant identity and tissues sampled are major driving factors for the endophytic fungal community composition and dynamics. Similar results on the microecological distribution of endophytes in different tissues in *Angelica sinensis* and *Azadirachta indica* were also observed, which also demonstrated that endophytes have tissue specificity [19,27]. The abundance, richness, species composition, and diversity of endophytic assemblages of *Z. bungeanum* were found to be significantly dependent on the sample tissue. Earlier studies have proposed that possible reasons for the diversity are the physiology and chemistry of the colonized tissues, different plant inhabitants or a different environment might influence endophyte recruitment [28,29].

All of endophytic fungi isolated from *Z. bungeanum* belonged mainly to the Ascomycota, within in the classes of Sordariomycetes and Dothideomycetes by morphological and ITS sequence identification. Other researchers also reported that Sordariomycetes and Dothideomycetes are the main groups of endophytic fungi from other plants [26,30]. Endophytic fungal species abundance distribution was widely reported to be skewed, with many frequent species and several incidental species, which might be related to the sampling size and method [20]. There is obvious tissue specificity of endophytic fungi genera in *Z. bungeanum*. Similar results have also been widely reported in other plants [7]. The genera of *Fusarium* and *Alternaria* are common in the stems, roots, leaves, fruits, and thorns of *Z. bungeanum*. The genera *Bionectria*, *Rosellinia*, *Paraphoma*, *Rhizopycnis*, and *Acrocalymma* were only isolated from the roots. Seven genera, *Nectria*, *Clonostachys*, *Sarocladium*, *Leptosphaerulina*, *Epicoccum*, *Botryosphaeria*, and *Irpex*, were specific to the stems. The genus *Auricularia* was only isolated from the leaves. Other genera can be isolated from two or three different tissues of *Z. bungeanum*. In the present study, *Alternaria*, *Fusarium*, and *Phoma* were frequently isolated species with high relative abundances of 30.85%, 13.71%, and 12.77%, respectively (Figure 3F), all of which have a cosmopolitan distribution and are found in association with a wide variety of host plants [31,32,33]. *Alternaria* and *Fusarium* are reported to be the most frequent and common genera of endophytic fungi from different plant species as well as various environmental conditions [21,34]. Several other genera were also isolated from *Z. bungeanum*, including *Gibberella*, *Nectria*, *Clonostachys*, *Bionectria*, *Phomopsis*, *Cytospora*, *Diaporthe*, *Rosellinia*, *Sarocladium*, *Peyronellaea*, *Leptosphaerulina*, *Epicoccum*, *Paraphoma*, *Rhizopycnis*, *Acrocalymma*, *Dothiorella*, *Botryosphaeria*, *Aspergillus*, *Irpex*, and *Auricularia*. Although several endophytic fungal genera were isolated with low relative abundance, those minor genera may have an important ecological role for their host plants or could be capable of synthesizing bioactive compounds [34]. Some of the above endophytic fungal genera are reported to be commonly associated with plant disease symptoms in several plants. For example, *F. proliferatum* is a common pathogen of numerous crops and an agent of wilt, blight, and diebacks of palm trees [35]. The *Diaporthe* and *Phomopsis* complex are causal agents of seed decay and cause soybean blight and canker diseases [36]. *Botryosphaeria* and its anamorph complex are especially responsible for symptoms such as fruit rot, shoot blight, dieback, and canker of numerous woody hosts [37]. However, it is not inconsistent that endophytic fungi could also be pathogens because plant pathogens and endophytes might convert mutually by influencing a favorable outer environment or plant disease conditions [38].

In recent years, there has been an increasing demand for identifying new antimicrobial agents due to the development of pathogen resistance to available pesticides. Although many chemically synthesized pesticides have been generated, their side effects have also been frequently reported, including pesticide residue, pathogenic resistance to pesticide, or resurgence of pests [4]. Endophytic fungi have been considered a novel resource of natural antimicrobial compounds with efficient and environmentally friendly characteristics [39,40]. It was observed that the ethyl acetate (EtOAc) extract of the endophytic fungus *Trichoderma harzianum* offered excellent control of the tomato gray mold caused by *Botrytis cinerea* without fungicide resistance and in an environmentally friendly manner [41]. Santiago et al. [42] also found one endophytic fungal isolate from *Cinnamomum mollissimum* that possessed efficient killing ability against the pathogenic fungus *Aspergillus niger*. Pan et al. [43] found that an EtOAc extract of the endophytic fungus *Chaetomium globosum* from *Houttuynia cordata* showed a wide antifungal spectrum. The specific secondary metabolites of endophytic fungi, such as helvolic acid, fumitremorgin B, verruculogen, and spirobisnaphthalenes, were also found to exhibit strong antifungal activity against multifarious plant pathogenic fungi [44,45,46]. In the present work, we investigated the inhibitory effects of the crude EtOAc extracts of endophytic fungi from *Z. bungeanum* on host-specific pathogenic fungi *F. sambucinum* and *P. zanthoxyli*. Several endophytic fungi were observed with inhibitory effects on both of the pathogens. However, there were only five endophytic isolates that showed obvious strong inhibitory effects (*IR* > 50%) on *F. sambucinum*: Zbf-S11 (*Epicoccum nigrum*), Zbf-S27 (*Diaporthe* sp.), Zbf-S47 (*Peyronellaea glomerata*), Zbf-S48 (*Phomopsis* sp.), and Zbf-S49 (*Phomopsis vaccinii*) (Figure 6A and Figure 7A). There were four endophytic fungal isolates that exhibited strong inhibitory effects on *P. zanthoxyli*: Zbf-S1 (*Fusarium* sp.), Zbf-S11 (*Epicoccum nigrum*), Zbf-R1 (*Fusarium* sp.), and Zbf-T3 (*Diaporthe cotoneastri*) (Figure 6B and Figure 7B). The endophytic isolate Zbf-S11 *E. Nigrum* has excellent inhibitory effects on both pathogens and is worth further investigation. Inhibitory rate dynamics of all eight endophytic fungi with strong inhibitory effects showed the characteristic of long-lasting efficiency, which might be attributed to the existence of antifungal compound produced by endophytic fungi. For example, two polyketides with prominent inhibitory activity were isolated from the endophytic fungus *Cryptosporiopsis* sp. obtained from *Zanthoxylum leprieurii* [47]. Antimicrobial fusaruside was characterized from the chloroform-methanol extract of endophytic *Fusarium* sp. IFB-121 of *Quercus variabilis* [48]. It is worth further isolating and characterizing secondary metabolites of *Z. bungeanum* endophytic fungi and establishing more bioactivity testing models to explore natural resources. It may be possible to utilize the endophytic fungi of *Z. bungeanum* as biocontrol agents to control its pathogenic fungi.

The present study is the first report to systematically analyze the biodiversity and antifungal activity of endophytic fungi isolated from *Z. bungeanum* using culture-dependent methods. This study demonstrated the tissue specificity of endophytic fungi in different parts of *Z. bungeanum.* All of the endophytic fungi of *Z. bungeanum* were identified by morphological observation and rDNA ITS identification. Moreover, the identification of endophytic fungi can be confirmed by the in-depth physiological metabolism, biochemical function detection, and sequence analyses of multiple gene regions in the future [49,50]. Using a culture-dependent method might miss some unculturable endophytic fungi, which might influence the endophytic fungi diversity results. Nevertheless, we have obtained numerous endophytic fungal isolates from *Z. bungeanum*. It is important to directly study the composition and structure of microbial populations at the genetic level by constructing the clone library and bypassing the step of strain isolation and plate cultivation, which is convenient, efficient, and more suitable for fungal diversity analysis due to the higher richness. We are carrying out the research using the method of clone libraries and rDNA ITS sequencing to systematically analyze the diversity of endophytic fungi of *Z. bungeanum*. It has also been reported that some genera might be excluded from clone libraries but could be isolated by pure cultivation [23]. The combination of the two methods would be complementary in achieving a better understanding of the diversity of fungal communities of *Z. bungeanum*. Eight endophytic fungal isolates from *Z. bungeanum*, especially Zbf-S11 *E. nigrum*, exhibited significant inhibitory effects on its host plant pathogenic fungi *F. sambucinum* and *P. zanthoxyli*. Although we only investigated the antifungal activity of the EtOAc extracts of endophytic fungi, we screened out several endophytic isolates with strong antifungal activity. If further experiments are carried out to isolate pure compounds and determine their biological activities, we might obtain many novel natural compounds with promising activity from endophytic fungi of *Z. bungeanum*. Presently, the systematical chemical analyses of secondary metabolites of the eight endophytic fungal isolates (Zbf-S11, Zbf-S27, Zbf-S47, Zbf-S48, Zbf-S49, Zbf-S1, Zbf-R1, Zbf-T3) are being carried out. The present research offers a framework for further investigation and utilization of endophytic fungi associated with *Z. bungeanum*.

## 4. Materials and Methods

### 4.1. Plant Material, Pathogenic Fungi and Chemicals

Ten healthy and asymptomatic three-year-old *Zanthoxylum bungeanum* (cultivar: Dahongpao) plants were randomly selected in July 2015, which covered the whole planting area of *Z. bungeanum* in the nursery garden of Northwest A&F University (34°16′ N; 108°4′ W) located in the Yangling District of Shaanxi province (China). The roots, stems, leaves, fruits, and thorns of each plant were collected and then immediately brought to the laboratory. Ten samples of every tissue from each plant were chosen randomly, and then we combined all samples of every tissue from 10plants. Finally, 100 samples of each tissue were obtained and stored at 4 °C in the refrigerator. All of the samples were used to isolate endophytic fungi within 24 h after collection.

Our research team obtained the pathogenic fungi *Fusarium sambucinum* and *Pseudocercospora zanthoxyli* in previous studies [16,17]. Both of these strains were maintained on potato dextrose agar (PDA) slants in cryovials at 4 °C.

All the chemicals were purchased from Jie Cheng Chemical and Glass Company (Yangling, China) except that those were peculiarly explained where they were bought.

### 4.2. Isolation and Preservation of Endophytic Fungi

All of the samples from each tissue were washed separately by running tap water to remove dust or other residues on the surface. For roots and stems, each sample was cut into approximate 1.0 cm × 1.0 cm segments by an autoclaved pinch cutter. For leaves, each sample was cut into a small disc with a diameter of approximately 1.0 cm. For fruits and thorns, each sample was cut with an incision. The samples from every tissue were disinfected by soaking in 75% ethanol for 2 min, then soaked in a 0.2% mercuric chloride solution for 10 min and then washed three times with autoclaved water. The 0.2% mercuric chloride disinfectant solution was recovered. After that, the samples were transferred onto dried sterile filter paper to remove the liquid from the surface of samples. Subsequently, each sample was placed on a potato dextrose agar (PDA) plate and kept at 25 °C in the incubator for 20 days. During the incubation period, all of the plant samples were observed every day, and any newly emerged fungal spot was immediately picked out by autoclaved toothpicks and transferred to another fresh PDA plate. The resulting fungal isolates were further purified and then maintained on PDA slants in cryovials at 4 °C and −80 °C. All operations were carried out under sterile conditions.

### 4.3. Identification of Endophytic Fungi

Initially, the purified isolates were grouped based on their morphological characteristics including colony color, hyphal shape and structure, growth rate, spore morphology and color, and exudatecolor. All of the endophytic fungi were first categorized according to their morphological characteristics. We defined the endophyic fungal isolates as being of the same morphotype if they possessed the same characteristics of colony, mycelia, and spore. In the study, 93 morphotypes were obtained. After that, one isolate representing one morphotype was selected for molecular identification. In our study, 93 isolates were then subjected to molecular identification by analyzing the internal transcribed spacer (ITS) region of the nuclear ribosomal DNA. All included steps were as follows: genomic DNA extraction, polymerase chain reaction (PCR) amplification, ITS sequencing, and analysis by basic local alignment search tool (BLAST).

Endophytic fungi were cultured on PDA plates before DNA extraction. When the colonies of endophytic fungi reached enough mass for DNA extraction, mycelia were scraped from the surface of the PDA plate using sterile toothpicks. Collected mycelia were ground into powder in liquid nitrogen using an autoclaved mortar. Then, 500 mg of mycelia powder was subjected to genomic DNA extraction using a TaKaRa MiniBEST Plant Genomic DNA Extraction Kit (Takara Biotechnology Co., Ltd., Dalian, China, Code No. 9768). The extraction process was carried out according to the manufacturer’s instructions. The extracted DNA was dissolved in 100 μL distilled water and stored at 4 °C until further use.

The total DNA was amplified by PCR using primers ITS1 (5′-TCCGTAGGTGAACCTGCGG-3′) and ITS4 (5′-TCCTCCGCTTATTGATATGC-3′) synthesized by Sangon Biotech Co., Ltd. (Shanghai, China) [43]. Amplification was conducted in 30 μL of PCR mixture containing 15 μL Premix TaqTM (Takara Biotechnology Co., Ltd., Dalian, China No. RR003A), 0.5μL template DNA, 1.0 μL ITS1 primer, 1.0 μL ITS4 primer, and 12.5 μL distilled water. PCR amplifications were performed in a thermal cycler with an initial denaturing step at 94 °C for 3 min, followed by 34 amplification cycles of 30 s denaturation at 94 °C, 30 s primers annealing at 54 °C, 45 s extension at 72 °C, and then a final elongation step of 10 min at 72 °C. PCR products were analyzed by electrophoresis using a 1% agarose gel (*w*/*v*) containing 0.01% (*v*/*v*) GoldView nucleic acid stain. Visual confirmation of the ITS region under the impact of UV light was performed by an image capture device.

All PCR products of endophytic fungi were sent to Sangon Biotech Co., Ltd. (Shanghai, China) for sequencing. The raw obtained sequences were aligned using MEGA7 [51], edited manually, and then BLAST (Basic Local Alignment Search Tool) was used to search for the best match in the National Center for Biotechnology Information (NCBI) GenBank database (http://www.ncbi.nlm.nih.gov/) to identify endophytic fungi. Sequences with similarity over 94% belonged to the same genus, and those with similarity over 97% belonged to the same species [23]. The consensus sequence data of 93 endophytic fungal isolates were summarized by SEQUIN and then submitted to NCBI and GenBank accession numbers were assigned (Table 1).

### 4.4. Phylogenetic Analyses of the Endophytic Fungi

Based on morphological and molecular identification results, the 93 endophytic fungal isolates in this study were classified into 14 families (Table 2). The endophytic fungi belonging to the same family were analyzed in the same phylogenetic tree. We selected a neighbor-joining (NJ) method to analyze the phylogenetic relationships of the *Z. Bungeanum* endophytic fungi. Each NJ tree was constructed by MEGA7 for the endophytic fungi belonging to the same family. The fungi used for each NJ tree alignment included tested endophytic fungi belonging to the family, several homologous fungal strains, and one exogenous fungal strain belonging to the same family but not the same genus. The ITS sequences of homologous and exogenous fungal strains were retrieved from NCBI. All sequence datasets were processed by MEGA7. The evolutionary history was inferred using the NJ method with 1000 Bootstrap replications. The phylogenetic tree was drawn to scale, with branch lengths in the same units as those of the evolutionary distance used to infer the phylogenetic tree. The evolutionary distances were computed using the Maximum Composite Likelihood method and are in the units of the number of base substitutions per site. All positions containing gaps and missing date were deleted. Finally, we constructed 14 NJ trees in our present research that were marked as family group A to N, successively.

### 4.5. Diversity Analysis of the Endophytic Fungi

Using species as the statistical unit, we counted the number of isolates (*N*) and calculated the isolation frequency (*IF*) for each endophytic fungal species in different tissues or the total plant (Table 3). The isolation frequency was calculated according to Equation (1). The species richness was evaluated by the species richness index (*S*) and Margalef index (*D*′), which are two important parameters for alpha diversity analysis [26]. Species richness index (*S*) was obtained by counting the number of endophytic fungal species in each tissue or total plant. The Margalef index (*D*′) was calculated by Equation (2).The species diversity was evaluated by the Shannon–Wiener index (*H*′), Simpson’s diversity index (*D*_s_), and Simpson’s dominant index (*λ*) [26,52]. The Shannon–Wiener index (*H*), Simpson’s diversity index (*D*_s_), and Simpson’s dominant index (*λ*) were calculated by Equations (3)–(5), respectively. The probability of interspecific encounter (*PIE*) index was used to evaluate the encountering probability of the individuals belonging to different species [53]. *PIE* index was calculated by Equation (6). Species Evenness was evaluated by Pielou’s evenness index (*J*) [34], which was calculated by Equation (7). The relative abundance (*RA*) for each genus was also calculated by Equation (8):

*IF*(%) = (*N*_i_/*N*_t_) × 100% (1)

*D*′ = (*S* − 1)/ln*N*_t_ (2)

(3) $$H\prime \;=\;-\sum_{i=1}^{s}P_{i}\ln P_{i},P_{i}\;=N_{i}/N_{t}$$

(4) $$D_{s}=\;1-\sum_{i=1}^{S}{P_{i}}^{2}$$

(5) $$\lambda =\sum_{i=1}^{S}{P_{i}}^{2}$$

(6) $$PIE=\sum_{i=1}^{s}（N_{i}/N_{t}）（N_{t}-N_{i}）/(N_{t}-1)$$

*J* = *H*/*H*_max_, *H*_max_= ln*S* (7)

*RA* (%) = *N*′/*N*_t_ ×100% (8)

where *N*_i_ is the number of isolates belonging to the *i*th species, *N*_t_ is the total number of endophytic fungal isolates in each tissue or total plant, *S* is the number of total species in each tissue or total plant, and *N*′ is the number of endophytic fungal isolates from each class, order, or genus in each tissue or total plant.

### 4.6. Metabolites Extraction from Endophytic Fungi

The metabolites of 93 endophytic fungal isolates, representing 93 morphotypes, were extracted by ethyl acetate (EtOAc) for antifungal assays. The endophytic fungi preserved at 4 °C in the refrigerator were separately inoculated on fresh PDA plates and then kept at 25 °C in an incubator for seven days. The endophytic fungal plug (5 mm diameter) of the mycelial inoculum was obtained by an autoclaved hole punch from the margin of an actively growing colony and then transferred into an Erlenmeyer flask (500 mL) containing 200 mL potato dextrose broth (three plugs per flask). All flasks were shaken in an incubator at 125 rpm at 25 °C for 14 days. The 14-day fermented broth cultures were extracted with 200 mL EtOAc three times. Each resulting EtOAc crude extract was collected and concentrated to dryness in a vacuum rotary evaporator at 40–45 °C and then washed with 3 mL EtOAc and transferred into a clean vial. Each empty vial was weighed before and after the EtOAc volatilized completely. The weight of EtOAc extract of each endophytic fungus was calculated.

### 4.7. Antifungal Assay for Endophytic Fungi

Antifungal activities of EtOAc extracts of 93 endophytic fungal isolates were carried out by colony radial mycelia growth method against pathogenic fungi *F. sambucinum* and *P. zanthoxyli* [54]. EtOAc extracts of each endophytic fungus were dissolved in 1 mL EtOAc and then filtered through a 0.22-μm Millipore filter. Each filtration was added into an Erlenmeyer flask (250 mL) containing 100 mL PDA before the PDA solidified and then mixed; the PDA medium was poured into sterile petri dishes (9 cm diameter). Each petri dish contained 10 mL PDA medium. The final concentrations of endophytic fungi EtOAc extracts in media are summarized in Table 3. The blank control was carried out without adding anything to the PDA media. The negative control was carried out with EtOAc addition into the PDA media (1%, *v*/*v*).

The pathogenic fungi *Fusarium sambucinum* and *Pseudocercospora zanthoxyli* were activated from dormant states by cultivation on PDA plates. After that, the pathogenic fungus plugs (5-mm diameter) from the margin of actively growing colonies were placed on the center of the PDA medium plates. Each treatment was carried out with five triplicates. All of the plates were then kept at 25 °C in the dark in an incubator for seven days, when the colony diameter was measured twice in perpendicular. The inhibitory rate (*IR*) was calculated by Equation (9):

*IR* (%) = {*D*_0_ − [*D*_s_ − (*D*_0_ − *D*_n_)]} × 100%/*D*_0_ (9)

where *D*_0_ is the average diameter of blank control, *D*_s_ is the average diameter of the treated sample, and *D*_n_ is the average diameter of the negative control.

## 5. Conclusions

The present study is the first to systematically investigate the biodiversity, phylogeny, and antifungal activity of endophytic fungi isolated from *Z. bungeanum*. High diversity and significant endophytic fungal tissue specificity were observed in *Z. bungeanum*. Phylogenetic analyses of the endophytic fungi can provide information for exploring the evolution and community dynamics of fungi residing in *Z. Bungeanum*. Endophytic fungi with obvious strong and long-lasting inhibition against host-specific phytopathogens were obtained from *Z. bungeanum*, which might be considered potential biological control agents for plant disease. Moreover, the study also reveals the value of sampling different tissues of a given plant to obtain the greatest endophyte species diversity, which can offer a framework for further investigation and utilization of endophytic fungi as the unique source of the interesting and useful bioactive compounds.

## Figures and Tables

**Table 1:** Identification of endophytic fungi from *Zanthoxylum bungeanum* by basic local alignment search tool (BLAST) in GenBank.

Morphotype	GenBank Access Number	BLAST Match Results				Sequence Length (bp)	Isolate Number (*N*)
		Closest Species	GenBank Access Number	Query Coverage (%)	Identity (%)		
Zbf-S1	KX064992	*Fusarium* sp.	EU818693	98	99	526	10
Zbf-S2	KX064993	*Epicoccum nigrum*	KM434160	99	99	520	4
Zbf-S3	KX064994	*Alternaria alternata*	KF380824	98	100	539	10
Zbf-S4	KX064995	*Clonostachys byssicola*	KC806270	96	99	546	3
Zbf-S5	KX064996	*Aspergillus flavus*	LN482520	98	99	570	2
Zbf-S6	KX064997	*Alternaria tenuissima*	JX867218	98	99	542	5
Zbf-S7	KX064998	*Irpex lacteus*	JX290571	99	99	646	3
Zbf-S8	KX064999	*Phoma glomerata*	FJ517547	99	99	518	4
Zbf-S9	KX065000	*Sarocladium implicatum*	KM520017	98	99	560	3
Zbf-S10	KX065001	*Fusarium proliferatum*	JQ693101	98	99	530	5
Zbf-S11	KX065002	*Epicoccum nigrum*	KT276979	95	100	526	4
Zbf-S12	KX065003	*Alternaria tenuissima*	KC337036	97	100	543	11
Zbf-S13	KX065004	*Fusarium fujikuroi*	KF572451	99	99	517	8
Zbf-S14	KX065005	*Fusarium solani*	JQ771182	96	99	544	12
Zbf-S15	KX065006	*Gibberella moniliformis*	JF499677	96	99	537	5
Zbf-S16	KX065007	*Leptosphaerulina australis*	KF293970	98	99	514	7
Zbf-S17	KX065008	*Dothiorella gregaria*	FJ517548	98	99	517	8
Zbf-S18	KX065009	*Nectria haematococca*	KF887043	97	99	539	8
Zbf-S19	KX065010	*Botryosphaeria dothidea*	KP183164	98	99	559	9
Zbf-S20	KX065011	*Alternaria alternata*	KJ526174	98	99	545	11
Zbf-S21	KX065012	*Epicoccum nigrum*	KM434167	99	99	522	5
Zbf-S22	KX065013	*Phomopsis* sp.	HQ832815	98	99	565	6
Zbf-S23	KX065014	*Diaporthe* sp.	KC763089	99	99	559	6
Zbf-S24	KX065015	*Cytospora* sp.	KJ463521	94	99	568	7
Zbf-S25	KX065016	*Diaporthe* sp.	KC763085	98	99	561	5
Zbf-S26	KX065017	*Phomopsis vaccinii*	KJ193729	98	99	563	4
Zbf-S27	KX065018	*Diaporthe* sp.	FJ175370	98	99	560	6
Zbf-S28	KX065019	*Diaporthe eres*	HQ115664	98	99	561	4
Zbf-S29	KX065020	*Phomopsis* sp.	HQ832815	98	99	546	7
Zbf-S30	KX065021	*Diaporthe* sp.	KR094451	97	99	565	5
Zbf-S31	KX065022	*Alternaria brassicae*	KT803065	98	99	542	7
Zbf-S32	KX065023	*Alternaria* sp.	KP211537	98	99	543	5
Zbf-S33	KX065024	*Dothiorella gregaria*	FJ517548	99	99	517	4
Zbf-S34	KX065025	*Phoma moricola*	KF293794	99	99	517	8
Zbf-S35	KX065026	*Phoma fungicola*	KF293764	100	99	514	4
Zbf-S36	KX065027	*Gibberella moniliformis*	JX970629	98	99	532	9
Zbf-S37	KX065028	*Phomopsis capsici*	KR870865	97	99	560	3
Zbf-S38	KX065029	*Phomopsis vaccinii*	KJ739493	97	97	562	4
Zbf-S39	KX065030	*Diaporthe nobilis*	KJ609006	97	99	557	5
Zbf-S40	KX065031	*Diaporthe* sp.	KC145882	97	99	556	6
Zbf-S41	KX065032	*Diaporthe* sp.	KC763084	96	99	558	5
Zbf-S42	KX065033	*Botryosphaeria dothidea*	KP183170	99	99	556	7
Zbf-S43	KX065034	*Phoma medicaginis*	KF293741	100	99	516	2
Zbf-S44	KX065035	*Peyronellaea pinodella*	KF293765	98	99	517	2
Zbf-S45	KX065036	*Peyronellaea* sp.	HQ914833	99	99	519	1
Zbf-S46	KX065037	*Peyronellaea glomerata*	KT192373	98	99	518	3
Zbf-S47	KX065038	*Peyronellaea glomerata*	KT192373	98	99	516	4
Zbf-S48	KX065039	*Phomopsis* sp.	JF812647	98	99	557	7
Zbf-S49	KX065040	*Phomopsis vaccinii*	KJ739493	97	99	559	6
Zbf-S50	KX065041	*Diaporthe nobilis*	KJ609006	97	99	556	7
**Total-S**							**286**
Zbf-R1	KX064979	*Fusarium* sp.	JQ929900	99	99	539	10
Zbf-R2	KX064980	*Rosellinia necatrix*	EF026117	98	99	559	15
Zbf-R3	KX064981	*Bionectria ochroleuca*	HQ385971	96	99	541	16
Zbf-R4	KX079482	*Fusarium solani*	KT366735	98	99	545	24
Zbf-R5	KX079483	*Fusarium solani*	KF494130	96	100	540	20
Zbf-R6	KX064982	*Rhizopycnis vagum*	JN859316	95	100	518	25
Zbf-R7	KX064983	*Alternaria* sp.	KF293773	100	99	544	30
Zbf-R8	KX064984	*Phoma fungicola*	KF293780	98	100	518	34
Zbf-R9	KX064985	*Fusarium solani*	KF494131	96	100	542	10
Zbf-R10	KX064986	*Aspergillus fumigatus*	KM520022	99	99	570	5
Zbf-R11	KX064987	*Acrocalymma vagum*	KP784427	98	99	519	27
Zbf-R12	KX064988	*Fusarium solani*	KT366735	99	99	544	15
Zbf-R13	KX064989	*Paraphoma* sp.	KJ655436	98	99	533	13
Zbf-R14	KX064990	*Phoma medicaginis*	KF293990	95	100	521	12
Zbf-R15	KX064991	*Fusarium solani*	KC156593	99	99	543	8
**Total-R**							**264**
Zbf-L1	KX064971	*Phoma medicaginis*	KF293990	99	99	516	25
Zbf-L2	KX064972	*Gibberella fujikuroi*	JQ363721	99	99	518	12
Zbf-L3	KX064973	*Auricularia polytricha*	FJ617294	96	99	544	27
Zbf-L4	KX079484	*Alternaria tenuissima*	KT310953	98	99	549	30
Zbf-L5	KX079485	*Dothiorella gregaria*	FJ517548	98	99	520	16
Zbf-L6	KX064974	*Alternaria* sp.	KF887100	96	99	550	10
Zbf-L7	KX064975	*Alternaria porri*	KR912293	96	99	549	15
Zbf-L8	KX079486	*Alternaria solani*	JN903925	97	99	546	14
Zbf-L9	KX064976	*Alternaria alternata*	KT218505	98	99	539	17
Zbf-L10	KX064977	*Alternaria tenuissima*	KU508797	97	99	542	16
Zbf-L11	KX064978	*Alternaria alternata*	KP900243	98	99	546	20
**Total-L**							**202**
Zbf-F1	KX064962	*Phoma medicaginis*	KF293990	97	99	521	9
Zbf-F2	KX064963	*Peyronellaea* sp.	HQ914833	99	99	514	9
Zbf-F3	KX064964	*Peyronellaea* sp.	JN986777	98	99	516	11
Zbf-F4	KX064965	*Dothiorella gregaria*	FJ517548	100	99	515	15
Zbf-F5	KX064966	*Alternaria alternata*	KF644350	97	99	542	20
Zbf-F6	KX064967	*Dothiorella gregaria*	FJ517548	99	99	515	11
Zbf-F7	KX064968	*Alternaria alternata*	KF644350	98	99	543	16
Zbf-F8	KX064969	*Alternaria tenuissima*	KC337036	98	100	541	8
Zbf-F9	KX064970	*Alternaria* sp.	KF293779	100	99	544	7
**Total-F**							**106**
Zbf-T1	KX065042	*Phoma fungicola*	KF293763	98	99	522	12
Zbf-T2	KX065043	*Phomopsis* sp.	HQ832675	95	99	558	8
Zbf-T3	KX065044	*Diaporthe cotoneastri*	KJ609015	97	98	567	7
Zbf-T4	KX065045	*Alternaria tenuissima*	KT310953	99	99	550	18
Zbf-T5	KX06504	*Phoma medicaginis*	KF293990	96	100	524	10
Zbf-T6	KX065047	*Fusarium proliferatum*	KF986664	94	99	536	7
Zbf-T7	KX079487	*Alternaria* sp.	HQ385970	98	100	548	16
Zbf-T8	KX065048	*Alternaria alternata*	KP278204	95	99	557	4
**Total-T**							**82**
**Total**							**940**

**Table 2:** Cluster analyses of all endophytic fungi isolates from *Z. bungeanum*.

Group	Morphotype	Taxa			
		Species	Family	Order	Class
A	S1,R1	*Fusarium* sp.	Nectriaceae	Hypocreales (19.68%)	Sordariomycetes (32.77%)
	S10, T6	*Fusarium proliferatum*			
	S13	*Fusarium fujikuroi*			
	S14, R4, R5, R9, R12, R15	*Fusarium solani*			
	S15,S36	*Gibberella moniliformis*			
	L2	*Gibberella fujikuroi*			
	S18	*Nectria haematococca*			
B	S4	*Clonostachys byssicola*	Bionectriaceae		
	R3	*Bionectria ochroleuca*			
C	S9	*Sarocladium implicatum*	Incertae sedis		
D	S22, S29, T2, S48	*Phomopsis* sp.	Valsaceae	Diaporthales (11.49%)	
	S26, S38, S49	*Phomopsis vaccinii*			
	S37	*Phomopsis capsici*			
	S24	*Cytospora* sp.			
E	T3	*Diaporthe cotoneastri*	Diaporthaceae		
	S23, S25, S27, S30, S40, S41	*Diaporthe* sp.			
	S28	*Diaporthe eres*			
	S39, S50	*Diaporthe nobilis*			
F	R2	*Rosellinia necatrix*	Xylariaceae	Xylariales (1.60%)	
G	R13	*Paraphoma* sp.	Phaeosphaeriaceae	Pleosporales (55.85%)	Dothideomycetes (63.30%)
H	R6	*Rhizopycnis vagum*	Morosphaeriaceae		
	R11	*Acrocalymma vagum*			
I	S6, S12, T4, L4, L10, F8	*Alternaria tenuissima*	Pleosporaceae		
	S32, R7, L6, F9, T7	*Alternaria* sp.			
	L7	*Alternaria porri*			
	L8	*Alternaria solani*			
	L9, L11, F5, F7, T8, S3, S20	*Alternaria alternata*			
	S31	*Alternaria brassicae*			
J	S8	*Phoma glomerata*	Didymellaceae		
	S34	*Phoma moricola*			
	S35, R8, T1	*Phoma fungicola*			
	T5, S43, R14, F1, L1	*Phoma medicaginis*			
	S44	*Peyronellaea pinodella*			
	S45, F2, F3	*Peyronellaea* sp.			
	S46, S47	*Peyronellaea glomerata*			
	S16	*Leptosphaerulina australis*			
	S2, S11, S21	*Epicoccum nigrum*			
K	S17, S33, L5, F4, F6	*Dothiorella gregaria*	Botryosphaeriaceae	Botryosphaeriales (7.45%)	
	S19, S42	*Botryosphaeria dothidea*			
L	S5	*Aspergillus flavus*	Aspergillaceae	Eurotiales (0.74%)	Eurotiomycetes (0.74%)
	R10	*Aspergillus fumigatus*			
M	S7	*Irpex lacteus*	Incertae sedis	Polyporales (0.32%)	Agaricomycetes (3.19%)
N	L3	*Auricularia polytricha*	Auriculariaceae	Auriculariales (2.87%)	

The values in percentage form in parentheses are the relative abundance (RA) values.

**Table 3:** Isolation frequency (*IF*) of each endophytic fungal species from *Z. bungeanum*.

Endophytic Fungal Species	Root		Stem		Leaf		Fruit		Thorn		Total	
	*N*	*IF*	*N*	*IF*	*N*	*IF*	*N*	*IF*	*N*	*IF*	*N*	*IF*
*Fusarium* sp.	10	3.79	10	3.50	--	--	--	--	--	--	20	2.13
*Fusarium solani*	77	29.17	12	4.20	--	--	--	--	--	--	89	9.47
*Fusarium proliferatum*	--	--	5	1.75	--	--	--	--	7	8.54	12	1.28
*Fusarium fujikuroi*	--	--	8	2.80	--	--	--	--	--	--	8	0.85
*Gibberella moniliformis*	--	--	14	4.90	--	--	--	--	--	--	14	1.49
*Gibberella fujikuroi*	--	--	--	0.00	12	5.94	--	--	--	--	12	1.28
*Nectria haematococca*	--	--	8	2.80	--	--	--	--	--	--	8	0.85
*Clonostachys byssicola*	--	--	3	1.05	--	--	--	--	--	--	3	0.32
*Bionectria ochroleuca*	16	6.06	--	0.00	--	--	--	--	--	--	16	1.70
*Diaporthe* sp.	--	--	33	11.54	--	--	--	--	--	--	33	3.51
*Diaporthe nobilis*	--	--	12	4.20	--	--	--	--	--	--	12	1.28
*Diaporthe eres*	--	--	4	1.40	--	--	--	--	--	--	4	0.43
*Diaporthe cotoneastri*	--	--	--	0.00	--	--	--	--	7	8.54	7	0.74
*Cytospora* sp.	--	--	7	2.45	--	--	--	--	--	--	7	0.74
*Phomopsis* sp.	--	--	20	6.99	--	--	--	--	8	9.76	28	2.98
*Phomopsis vaccinii*	--	--	14	4.90	--	--	--	--	--	--	14	1.49
*Phomopsis capsici*	--	--	3	1.05	--	--	--	--	--	--	3	0.32
*Rosellinia necatrix*	15	5.68	--	0.00	--	--	--	--	--	--	15	1.60
*Epicoccum nigrum*	--	--	13	4.55	--	--	--	--	--	--	13	1.38
*Alternaria* sp.	30	11.36	5	1.75	10	4.95	7	6.60	16	19.51	68	7.23
*Alternaria alternata*	--	--	21	7.34	37	18.32	36	33.96	4	4.88	98	10.43
*Alternaria tenuissima*	--	--	16	5.59	46	22.77	8	7.55	18	21.95	88	9.36
*Alternaria porri*	--	--	--	0.00	15	7.43	--	--	--	--	15	1.60
*Alternaria solani*	--	--	--	0.00	14	6.93	--	--	--	--	14	1.49
*Alternaria brassicae*	--	--	7	2.45	--	--	--	--	--	--	7	0.74
*Phoma medicaginis*	12	4.55	2	0.70	25	12.38	9	8.49	10	12.20	58	6.17
*Phoma fungicola*	34	12.88	4	1.40	--	--	--	--	12	14.63	50	5.32
*Phoma glomerata*	--	--	4	1.40	--	--	--	--	--	--	4	0.43
*Phoma moricola*	--	--	8	2.80	--	--	--	--	--	--	8	0.85
*Peyronellaea* sp.	--	--	--	0.35	--	--	20	18.87	--	--	21	2.23
*Peyronellaea glomerata*	--	--	7	2.45	--	--	--	--	--	--	7	0.74
*Peyronellaea pinodella*	--	--	2	0.70	--	--	--	--	--	--	2	0.21
*Acrocalymma vagum*	27	10.23	--	0.00	--	--	--	--	--	--	27	2.87
*Paraphoma* sp.	13	4.92	--	0.00	--	--	--	--	--	--	13	1.38
*Dothiorella gregaria*	--	--	12	4.20	16	7.92	26	24.53	--	--	54	5.74
*Botryosphaeria dothidea*	--	--	16	5.59	--	--	--	--	--	--	16	1.70
*Aspergillus flavus*	--	--	2	0.70	--	--	--	--	--	--	2	0.21
*Aspergillus fumigatus*	5	1.89	--	0.00	--	--	--	--	--	--	5	0.53
*Leptosphaerulina australis*	--	--	7	2.45	--	--	--	--	--	--	7	0.74
*Sarocladium implicatum*	--	--	3	1.05	--	--	--	--	--	--	3	0.32
*Rhizopycnis vagum*	25	9.47	--	0.00	--	--	--	--	--	--	25	2.66
*Irpex lacteus*	--	--	3	1.05	--	--	--	--	--	--	3	0.32
*Auricularia polytricha*	--	--	--	0.00	27	13.37	--	--	--	--	27	2.87
Total	264	100	286	100	202	100	106	100	82	100	940	100

“--” means the corresponding endophytic fungal species was not observed. *N* represents the isolation number of each endophytic fungal species.

**Table 4:** Diversity analyses of endophytic fungi from *Z. bungeanum*.

Diversity Index	Different Tissues					Total
	Root	Stem	Leaf	Fruit	Thorn	
Species richness (*S*)	11	32	9	6	7	43
Margalef index (*D*′)	1.7934	5.4809	1.5071	1.0722	1.3616	6.1351
Shannon–Wiener index (*H*′)	2.1476	3.2010	2.0709	1.6100	1.9841	3.2743
Simpson diversity index (*D_s_*)	0.8528	0.9502	0.8588	0.7716	0.8510	0.9476
PIE index (*PIE*)	0.8561	0.9536	0.5567	0.7790	0.8615	0.9486
Dominant index (*λ*)	0.1472	0.0498	0.1412	0.2284	0.1490	0.0524
Pielou index (*J*)	0.8956	0.9236	0.9425	0.8986	1.0196	0.8705

**Table 5:** Concentrations of crude extracts of 93 endophytic fungal isolates in antifungal assays against pathogenic fungi *F. sambucinum* and *P. zanthoxyli*.

Isolates	Concentrations (mg/mL)	Isolates	Concentrations (mg/mL)	Isolates	Concentrations (mg/mL)
Zbf-R1	0.058	Zbf-S17	0.003	Zbf-S48	0.214
Zbf-R2	0.022	Zbf-S18	0.021	Zbf-S49	0.247
Zbf-R3	0.028	Zbf-S19	0.006	Zbf-S50	0.016
Zbf-R4	0.024	Zbf-S20	0.232	Zbf-L1	0.044
Zbf-R5	0.045	Zbf-S21	0.027	Zbf-L2	0.021
Zbf-R6	0.038	Zbf-S22	0.055	Zbf-L3	0.030
Zbf-R7	0.050	Zbf-S23	0.302	Zbf-L4	0.025
Zbf-R8	0.036	Zbf-S24	0.103	Zbf-L5	0.024
Zbf-R9	0.025	Zbf-S25	0.086	Zbf-L6	0.024
Zbf-R10	0.020	Zbf-S26	0.055	Zbf-L7	0.024
Zbf-R11	0.196	Zbf-S27	0.498	Zbf-L8	0.026
Zbf-R12	0.025	Zbf-S28	0.235	Zbf-L9	0.027
Zbf-R13	0.032	Zbf-S29	0.038	Zbf-L10	0.026
Zbf-R14	0.025	Zbf-S30	0.149	Zbf-L11	0.026
Zbf-R15	0.032	Zbf-S31	0.013	Zbf-F1	0.025
Zbf-S1	0.100	Zbf-S32	0.099	Zbf-F2	0.027
Zbf-S2	0.024	Zbf-S33	0.014	Zbf-F3	0.022
Zbf-S3	0.032	Zbf-S34	0.029	Zbf-F4	0.025
Zbf-S4	0.073	Zbf-S35	0.013	Zbf-F5	0.024
Zbf-S5	0.027	Zbf-S36	0.015	Zbf-F6	0.023
Zbf-S6	0.008	Zbf-S37	0.094	Zbf-F7	0.021
Zbf-S7	0.230	Zbf-S38	0.130	Zbf-F8	0.028
Zbf-S8	2.020	Zbf-S39	0.055	Zbf-F9	0.025
Zbf-S9	0.101	Zbf-S40	0.237	Zbf-T1	0.051
Zbf-S10	0.018	Zbf-S41	0.065	Zbf-T2	0.073
Zbf-S11	0.169	Zbf-S42	0.128	Zbf-T3	0.090
Zbf-S12	0.190	Zbf-S43	0.018	Zbf-T4	0.031
Zbf-S13	0.230	Zbf-S44	0.147	Zbf-T5	0.038
Zbf-S14	0.014	Zbf-S45	0.016	Zbf-T6	0.154
Zbf-S15	0.010	Zbf-S46	0.025	Zbf-T7	0.053
Zbf-S16	0.268	Zbf-S47	0.363	Zbf-T8	0.026

*F. sambucinum* was treated with the same concentration of crude extracts of endophytic fungi as *P. zanthoxyli*.

## Abbreviations

BLAST	Basic local alignment search tool
EtOAc	Ethyl acetate
IF	Isolation frequency
IR	Inhibitory rate
ITS	Internal transcribed spacer
NJ	Neighbor-joining
OTU	Operational taxonomic unit
PCR	Polymerase chain reaction
PIE	Probability of interspecific encounter
RA	Relative abundance
